# Indirect costs associated with ulcerative colitis: a systematic literature review of real-world data

**DOI:** 10.1186/s12876-019-1095-9

**Published:** 2019-11-09

**Authors:** Joelle Constantin, Petar Atanasov, Daniel Wirth, Andras Borsi

**Affiliations:** 1 Amaris, Barcelona, Spain; 2 Janssen-Cilag GmbH, Neuss, Germany; 3 Janssen EMEA, High Wycombe, UK

**Keywords:** Inflammatory bowel disease, Ulcerative colitis, Indirect costs, Productivity loss, Financial burden, Real world evidence, Systematic literature review

## Abstract

**Background:**

The economic burden of ulcerative colitis (UC), specifically related to indirect costs, is not extensively documented. Understanding and quantifying it is required by health care decision makers.

**Aim:**

To assess the impact of indirect costs of UC in observation studies.

**Method:**

A systematic literature search was conducted in MEDLINE®, Embase® and Cochrane Library to capture all relevant publications reporting outcomes on absenteeism, presenteeism and productivity losses in moderate to severe UC. Eligibility criteria for inclusion into the review were established using a predefined PICOS scheme. All costs were adjusted to 2017 currency values (USD dollars, $).

**Results:**

In total, 18 studies reporting data on indirect costs were included in the analysis. Absenteeism costs were classified into three categories: sick leave, short-term and long-term disability. Most of the studies captured absenteeism costs related specifically to sick leave, which was experienced on average by 10 to 24% patients with UC. Only three studies captured presenteeism costs, as these are difficult to measure, however costs ranged from 1602 $ to 2947 $ per patient year. The proportion of indirect costs accounted for 35% of total UC costs (Total UC costs were defined as the sum of healthcare costs, productivity costs and out-of-pocket costs).

**Discussion:**

A limited number of studies were identified describing the indirect costs in patients with moderate to severe UC. Insufficient data on different components of costs allowed a limited analysis on the impact of indirect costs in patients with UC. Further studies are needed to gain an understanding of the influence of UC on patients’ functional abilities.

## Background

Ulcerative colitis (UC) is a chronic disease that is characterized by diffuse mucosal inflammation limited to the colon [1, 2]. In about 95% of cases, UC affects the rectum and may extend to involve part or all of the large intestine. The clinical course of the disease is marked by episodes of exacerbations and remissions, occurring spontaneously or in response to treatment changes [3]. In spite of recent advances in therapy, the clinical burden and morbidity associated with UC remain high and may result in social and psychological sequelae if poorly controlled [4].

The management of UC has changed with the approval of new biological therapies such as infliximab, which was approved by the FDA (2005) and by the EMA (2006) for the treatment of moderate to severe UC [5, 6].

Apart from the clinical repercussions associated with this disease, UC also has a societal burden on patients and their caregivers. On a global scale, this disorder accounts for a quarter million physician visits, 30,000 hospitalizations, and more than a million workdays missed annually [7]. The direct medical costs alone exceed 4 billion dollars (USD) annually and are driven mainly by hospitalization events [7]. The economic burden of UC, specifically related to indirect costs, has not been extensively documented. As indirect costs account for a significant percentage of total UC costs, understanding and quantifying the economic burden of UC is required by health care systems to control and avoid costs associated to productivity losses in a societal perspective [7, 8].

vConclusions drawn from clinical trials are not always sufficient for decision makers, as they assess the value of a specific drug in a controlled setting. However, real word studies that collect data beyond Phase III controlled trials (i.e. under real life practice) allow decision makers to better manage and understand uncertainties, specifically related to epidemiology, compliance, adherence and cost insights [9]. The aim of this systematic literature review (SLR) was to assess the impact of indirect costs of UC, especially related to surgery and to the use of biologic therapies in real world.

## Materials and methods

### Indirect costs

As previously defined in the publication by Kawalec et al. [10] indirect costs (or productivity losses) are the labor earnings that are forgone as a result of an adverse health outcome. A decrease in productivity can result in illness, early death, side effects, or even time spent receiving treatment. Indirect costs can be categorized into three major components: (1) absence from paid work including sick leave, early retirement and reduced employment or unemployment (absenteeism), (2) reduced productivity of paid work (presenteeism), and (3) reduced opportunities for unpaid activities (loss of leisure) [11].

### Literature search

This review was conducted to identify studies that report indirect costs in ulcerative colitis. The protocol for this review was not registered.

The electronic databases Embase®, MEDLINE® and Cochrane Library were searched on the 22nd of May 2017 to capture studies reporting outcomes on absenteeism, presenteeism and productivity losses. Search terms included the following medical subject headings ‘ulcerative colitis’/exp. OR ‘ulcerative colitis’ OR ‘inflammatory bowel disease’/exp. OR ‘inflammatory bowel disease’ OR ‘ibd’ as well as cost subject headings such as ‘indirect’ OR ‘productivity’ OR ‘economic’ OR ‘cost’ OR ‘loss’ OR ‘burden’ OR ‘human capital’. Additional hand searches were performed to identify studies published in important medical societies such as United European Gastroenterology Week (UEGW), European Crohn’s and Colitis Organization (ECCO) and Digestive Disease Week (DDW).

For inclusion in this review, studies needed to fulfill specific criteria in accordance with a predefined PICOS^1^ scheme [12],

I) **Population:** Adult patients with a confirmed diagnosis of active^2^ moderate to severe UC were considered as the target population.
II) **Intervention/ Comparators:** No restrictions were applied to these two parameters.
III) **Outcomes:** indirect costs or productivity losses associated with absenteeism and/or presenteeism were considered of interest.
IV) **Study type:** Real Word Evidence (RWE) data, meaning observational studies, systematic literature reviews, cost estimation studies and cost effectiveness studies written in English and published between 1st of January 2006 (availability of anti-tumor necrosis factor drugs) and 22nd of May 2017 were included.

A publication was excluded if it did not fulfil the abovementioned inclusion criteria, meaning if the study did not report any kind of indirect costs or if the patient population did not have confirmed moderate to severe active UC. Randomized controlled trials, (i.e. phase 1, 2 and 3 studies), long-term extensions of clinical trials and studies with rules of protocol violation, as well as crossover trials and interventional cohort studies beyond the scope of public health interventions were excluded from this review. Publications that were not written in English were also excluded.

To assess the eligibility of a study, two authors independently examined titles and abstracts identified from the search strategy. Articles, which have been identified as potentially relevant based on title and abstract, were then reviewed in full text and selected according to the list of pre-specified inclusion/exclusion criteria. All discrepancies were solved by discussion. If no agreement was found, a third reviewer was involved in the discussion and final decision making.

As most of the studies extend across a wide time frame, all costs were converted to 2017 American dollars ($), using country specific consumer price index from the worldwide inflation data source.

This review was conducted in accordance with the Preferred Reporting Items for Systematic Reviews and Meta-Analyses (PRISMA) statement to ensure that all records were well tracked [13].

### Data extraction and synthesis of literature

Data from the eligible studies were collected, including publication details, specifications of the study question (indication, geographical scope, intervention, comparators and study objectives), methodology used, main indirect costing results (absenteeism specifically related to sick leave as well as short/long term disability and presenteeism costs), as well as limitations associated to the study. Results were then tabulated and analyzed using descriptive statistics. Data extraction was carried out by three researchers and quality control has been done for at least 20% of extracted data, as defined in the study protocol.

In order to achieve comparability of the results across publications, we attempted to break down indirect costs into the same categories (e.g. sick leave or short- and long-term disability). We also reported cost data per patient per year and assumed that costs are stable throughout the year.

### Statistical analysis

Descriptive statistics were reported for the study outcomes for all patients to describe the basic features of the data in the identified studies. Univariate, bivariate and multivariate analyses were not conducted to assess the association of baseline characteristics with cohorts nor patient characteristics with indirect costs.

## Results

### Literature search results

**Fig. 1:**
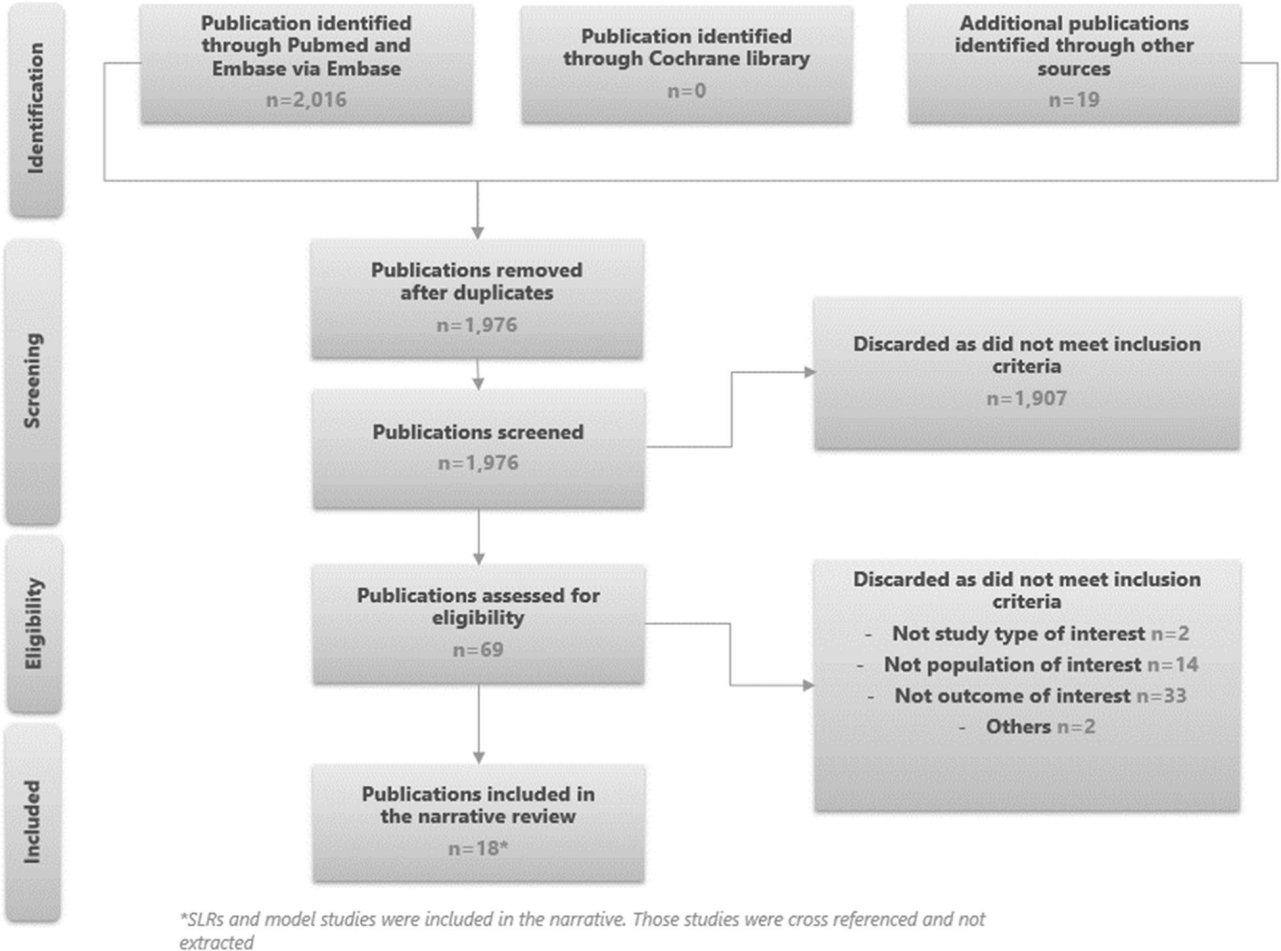
PRISMA chart for economic burden SLR

After having applied a search strategy for indirect costs in UC, 18 studies were incorporated in the narrative review, as outlined in Fig. 1. Most studies were conducted either in Europe or in the USA (12 studies in Europe, four studies in the USA, one study in Israel and one study did not report a country).

Data on indirect costs were collected from web-based questionnaire or through database analysis (17 studies), one study collected data through patient cost diaries. The majority of studies had large sample sizes, ranging from 53 to 6900 patients, as reported in Table 1.

**Table 1:** Characteristics of identified studies

Author, year	Country	Study population	Characteristics of study population	Follow up duration
Kawalec et al. 2017 [14]	Poland	Patients with UC	*N* = 202 Disease duration 26.35 years	October 2015- Q1 2016
Van Der Valk et al. 2016 [15]	The Netherlands	IBD patients	*N* = 566 Disease duration 16 years	2 years
Malinowski et al. 2016 [16]	Poland	UC patients	*N* = 6900	NR
Aldeguer et al. 2016 [17]	Spain	Patients with UC	*N* = 285	12 months
Cohen et al. 2015 [18]	USA	UC patients	*N* = 1728	2 years (1 year baseline period and 1 year study period)
Van Der Valk et al. 2014 [19]	The Netherlands	IBD Patients	*N* = 937 Disease duration 13.3 years	3 months
Meek et al. 2013 [20]	USA	Patients with UC	*N* = 31,050	12 months
Cohen et al. 2012 [21]	USA	UC patients	*N* = 1754	2 years
Kostic et al. 2015 [22]	Serbia	Patients with UC	*N* = 53	NR
Van Der Valk et al. 2012 [23]	The Netherlands	Patients with IBD	*N* = 928	2 years
Cannon et al. 2011 [24]	NR	IBD patients	*N* = 72	NR
Gibson et al. 2008 [25]	USA	Patients with CD and UC	*N* = 8970	NR
Mandel M et al. 2014 [1]	Hungary	IBD patients	*N* = 183 Disease duration 9.9 years	NR
Katz Avitan et al. 2016 [2]	Israel	UC patients	*N* = 150	NR
Hellström et al. 2017 [26]	Sweden	UC patients	*N* = 1361	2 years
Neovius et al. 2010 [27]	Sweden	UC patients	*N* = 2914	NR
Stark et al. 2006 [28]	Germany	UC patients	*N* = 483 Disease duration 12 years	NR
Van Der Valk et al. 2015 [29]	The Netherlands	IDB patients	Pouch: *n* = 81 Mean age 46.7 Disease duration 15 years	For 2 years at 3 months intervals
			Ileostomy: *n* = 48 Disease duration 18 years	
			Anti-TNFα: *n* = 34 Disease duration 16 years	

Overall, 13 studies out of 18 studies reported data from nine different countries (mainly in Europe), on absenteeism in UC, capturing cost/loss of earning of absenteeism per patient year and/or total average indirect costs of absenteeism per patient year. With regards to presenteeism, we were only able to identify three studies, one conducted in Poland [14], one in Hungary [1] and one in Israel [2]. Four studies assessed total productivity loss, two in The Netherlands [15, 19], one in Serbia [22] and one in Poland [14]. Both presenteeism and productivity losses reported cost per patient year, in USD dollars converted to 2017 base year. Furthermore, treatment allocation was not clearly defined in most publications. Only one study by Van der Valk et al. [29] focused on indirect costs of anti-TNF patients compared to patients who underwent surgery.

Throughout the identified studies, we noted that absenteeism costs were often classified into categories; either (1) costs associated to sick leave (Table 3), and/or (2) costs associated to short term and/or long term disability (Table 4) and/or (3) costs associated to absenteeism with no clear definition on the costing element mentioned (Table 2). These categories were analyzed independently and were reported in the sections below:

**Table 2:** Indirect costs associated with absenteeism in UC

Author, year	Country	% of patients experiencing absenteeism	Number of days absent per year/patient	Cost		Cost adjusted	
				Average cost absenteeism per patient year	Currency, base year	Average cost absenteeism per patient year	Currency, base year
Aldeguer et al. 2016 [17]	Spain	NR	1.08 days per year^a,b^	88.21^a^	EURO, 2012	105.40	$, 2017
Cohen et al. 2015 [18]	USA	98.20%	11.5 days per year^a^	3071	$, 2013	3196.60	$, 2017
Cohen et al. 2012 [21]	USA	NR	14.2 days per year^a^	NR	$, 2010	NR	$, 2017
Cannon et al. 2011 [24]	NR^d^	NR	60 days per year^c^	NR	NR	NR	NR
Gibson et al. 2008 [25]	USA	85.75%	NR	6020.50^e^	$, 2005	7793.50	$, 2017

NB: The publication by Katz Avitan et al. [2] conducted in Israel (not reported in the table) demonstrated that 21% of UC patients (*N* = 150) were experimenting absenteeism (defined as percentage of impairment while working)

^a^Due to medical visit specifically

^b^In the study it was reported as 29.55 h per year

^c^In the study, they reported that UC patient had missed more than 5 days from work in the past month

^d^This publication was an abstract and did not report the country where the study was conducted

^e^Mean absence costs, all employees, with or without claims

### Absenteeism cost

While some publications specifically define the components associated with absenteeism, there were few publications such as Cannon et al. [24] that did not have a clear definition of absenteeism, as reported in Table 2.

The proportion of absenteeism in ulcerative colitis patients was reported in two different countries the USA and Spain. The percentage of patients experiencing absenteeism was high, even if data was limited, ranging from 85 to 98% as reported by Gibson et al. [25] and Cohen et al. [18], respectively. This can be explained by the different types of population included in both the studies. Cohen et al. [18] includes moderate to severe UC patients treated with biologics, immunosuppressant or systemic corticosteroids during the first year study period whilst Gibson et al. [25] includes all types of patients diagnosed with UC.

The average number of absenteeism days per patient year varied widely, ranging from 1.08 to 60 days per patient year. The low rate of 1.08 days reported in Spain by Aldeguer et al. [17] is related to medical visits costs, where patients would be absent for a couple hours and would then return to work afterwards. Regarding the high rate of 60 days reported in Cannon et al. [24] (abstract), it was mentioned that only 9% (i.e. 7 patients) of UC patients experienced 60 days of absenteeism days per year. Without considering these two outliers, the range seems more stable across studies with a minimum of 11.5 of absenteeism days to a maximum of 14.2 absenteeism days per patient year in the USA.

The identified publications also report costs associated to absences from work ranging from 3197 $ to 7794 $ per patient year. Here again, the study by Aldeguer et al. [17] reported a lower value than the range (i.e. 105 $ per patient year), as seen in Table 2.

### Absenteeism – sick leave

Eight studies out of 18, all conducted in Europe, reported absenteeism costs related specifically to sick leaves in UC, as shown in Table 3. The type of patients in all these studies were pooled UC patients (i.e. with different disease states). Only Van der Valk et al. [29] looked at three types of UC patients, either treated with anti-TNFs, colectomy with J-pouch patients or colectomy with ileostomy patients. The percentage of patients experiencing sick leave was consistent across all studies and was estimated around 10 to 24%. However, the number of days missed from work due to sick leaves varied greatly between publications, more specifically between 8 to 63 days per patient year. In one study conducted in the Netherlands, it was shown that patients treated with anti-TNF therapies are more likely to be absent from work (28 days per patient year), compared to patients who underwent colectomy with J-pouch (10 days per patient year) and colectomy with ileostomy (20 days per patient year). This was also reflected when looking at the cost/ loss of earning (i.e. average cost of sick leave) 4824 $, 2147 $, and 1676 $, per patient year for anti TNF, pouch and ileostomy groups, respectively. The anti-TNF group accounted for the highest absenteeism cost compared to patients who underwent pouch and ileostomy surgery.

**Table 3:** Indirect costs associated with absenteeism, specifically sick leave in UC

Author, year	Country	Patients’ type	% of patients experiencing sick leave	Number of sick leaves days per patient per year	Cost			Cost adjusted		
					Average cost (€) of sick leave per patient per year	Total average productivity loss in Euros per year	Currency, base year	Average cost (€) of sick leave per patient per year	Total average productivity loss in Euros per year	Currency, base year
Mandel et al. 2014 [1]	Hungary	Pooled UC patients	NR	10.14^a^	485	1795	EURO, 2013	506	1872	$, 2017
Malinowski et al. 2016 [16]	Poland	Pooled UC patients	NR	13.49	787^e^	1260^e^	EURO, 2012	823	1318	$, 2017
Aldeguer et al. 2016 [17]	Spain	Pooled UC patients	22%	26.17	311	399	EURO, 2012	326	418	$, 2017
Van der Valk et al. 2012 [23]	The Netherlands	Pooled UC patients	NR	NR	1912^d^	NR	EURO, 2010	2401.6	NR	$, 2017
Van der Valk et al. 2014 [19]	The Netherlands	Pooled UC patients	13.4%^b^	10^cg^	1447 ^kg^	1580^g^	EURO, 2011	1608.4	1756	$, 2017
Van der Valk et al. 2015 [29]	The Netherlands	Pouch patients	10.50%	8^h^	1932^h^	NR	EURO, 2011	2147.5	NR	$, 2017
Van der Valk et al. 2015 [29]	The Netherlands	Ileostomy patients	13.50%	20^i^	1508^i^	NR	EURO, 2011	1676.2	NR	$, 2017
Van der Valk et al. 2015 [29]	The Netherlands	Anti-TNF patients	23.90%	28^j^	4340^j^	NR	EURO, 2011	4824.1	NR	$, 2017
Kawalec et al. 2017 [14]	Poland	Pooled UC patients	NR	30.49	679	2043	EURO, 2014	710.6	2138	$, 2017
Hellström et al. 2017 [26]	Sweden	Pooled UC patients	NR	63^f^	5309^f^	NR	EURO, 2014	5391	NR	$, 2017

^a^Missed working hours: The study reports missed working hours of 1.56 h per week equivalent of 10.14 days per year

^b^Unpaid work: 28; 3.0%

^c^Unpaid work: 7.8 days per 3 months

^d^The study reports 478€ per 3 month

^e^Gross domestic product per capita

^f^The study reports 50,925 SEK per year equivalent to either 5338 euros or 6297€ and it has been reported in the study it was reported as 9 weeks per year

^g^The study reports 2.5 days per 3 months and 361.79€ per 3 month

^h^The study reports 2 days per 3 months and 483€ per 3 month

^i^The study reports 5 days per 3 months and 377€ per 3 month

^j^The study reports 7 days per 3 months and 1085€ per 3 month

^k^Unpaid work: 18.33€ per 3 months

### Absenteeism – short- and long-term disability

Five [1, 16, 18, 25, 28] out of 18 studies reported data on short and/or long-term disability^3^ in patients with UC, of which two were conducted in the USA, one in Germany, one in Hungary and one in Poland, as can be seen in Table 4. The proportion of patients experiencing short term disability was reported in three studies (USA [18, 25] and Germany [28]) and was consistently ranging from 15 to 17%. Regarding long term disability, 7% of UC patients experienced it, as reported by Stark et al. [28], conducted in Germany, with a total number of disability days ranging from 10 to 12 per patient year. Regarding cost data, we found that authors distinguished between short- and long-term disability costs. As expected, on average long-term disabilities were more costly than short-term disabilities, ranging from 116 $ to 3019 $ per patient year for short term disability compared to the range of 1573 $ to 4394 $ per patient year for long term disability.

**Table 4:** Indirect costs associated with absenteeism- disability in UC

Author, year	Country	% of patients experiencing short term disability	% of patients experiencing long term disability	N. of days absent per patient per year	Costs				Costs adjusted			
					Average ST cost of disability per patient/year	Average LT cost of disability per patient/year	Average cost of productivity loss due to disability per patient/year	Currency, base year	Average ST cost of disability per patient/year	Average LT cost of disability per patient/year	Average cost of productivity loss due to disability	Currency, base year
Stark et al. 2006 [28]	Germany	15%	7%	NR	2912	4238	NR	EURO, 2004	3019	4394	NR	$, 2017
Cohen et al. 2015 [18]	USA	16.70%	NR	12.3	2713	2713	NR	$, 2013	2824	2824	NR	$, 2017
Gibson et al. 2008 [25]	USA	15.36%	NR	NR	1386	NR	NR	$, 2005	1794	NR	NR	$, 2017
Mandel et al. 2014 [1]	Hungary	NR	NR	10.14^be^	NR	NR	1310	EURO, 2013	NR	NR	1367	$, 2017
Malinowski et al. 2016^c^ [16]	Poland	NR	NR	NR	111	1503	NR	EURO, 2012	116	1573	NR	$, 2017

^a^It was reported as 224€ per month per short term and 326€ per month per long term disability

^b^Not only days associated to disability days

^c^Authors also estimated the average cost of disability in the long term for an unlimited period, 211€ however these patients are only a small part 6 out of 6900

^d^The study reported 1.56 h per week

### Presenteeism

Three studies estimated indirect costs associated to presenteeism, one in Poland [14], one in Hungary [1] and one in Israel [2], as indicated in Table 5.

**Table 5:** Indirect costs associated with presenteeism in UC

Author, year	Country	% of patients experiencing presenteeism	On the job productivity loss (%) due to UC per patient per week	Cost		Cost adjusted	
				Total indirect cost for presenteeism per patient per year	Currency, base year	Total indirect cost for presenteeism per patient per year	Currency, base year
Kawalec et al. 2017 [14]	Poland	55.20%	19.38%	1346	EURO, 2014	1602.1	$, 2017
Mandel et al. 2014^a^ [1]	Hungary	NR	19.42%	2410	EURO, 2013	2947.2	$, 2017

NB: The publication by Katz Avitan et al. [2] conducted in Israel (not reported in the table) demonstrated that 19% of UC patients (*N* = 150) experienced presesenteeism

^a^The study by Mandel et al. [1] reported 7.77 h lost due to presentism per patient per week. Value was converted using a 40 h labor week

UC patients were poorly described in the publication by Kawalec et al. [14] and in Katz Avitan et al. [2], however, more information on patient characteristics was provided in Mandel et al. [1]. In fact, it was reported that 11.5% of UC patients were on biologics and 8.3% of patients had underwent colectomy.

Consistent results were found when comparing presenteeism data, as all reported that patients with UC experience a 19% job productivity loss per week, with presenteeism costs ranging from 1602 $ to 2947 $ per patient year.

### Total productivity loss

Total productivity losses were reported in four studies, one in Poland [14], one in Serbia [22] and two in the Netherlands [15, 19], as shown in Table 6. The proportion of indirect costs associated to total UC costs^4^ was reported in two studies, conducted in the Netherlands and by the same author. On average, indirect costs accounted for 35% of total UC costs. Furthermore, total productivity losses for high-income countries ranged from 1459 $ in the Netherland to 2431 $ in Poland per patient year, with costs being more expensive in Poland (2431 $) compared to the Netherlands (2058 $). However, in Serbia, a middle-income country, total productivity losses were lower (i.e. 1567 $ per patient year). This difference can be explained by the under-utilization of biologic therapy and low health services prices used by IBD patients in Serbia [22].

**Table 6:** Total productivity losses associated to UC

Author, year	Country	Proportion of indirect cost associated to total UC costs	Cost		Cost adjusted	
			Total productivity losses per patient year	Currency, base year	Total productivity losses per patient year	Currency, base year
Kostic et al. 2015 [22]	Serbia	NR	142,267	RSD,2015^b^	1567.0	$, 2017
Kawalec et al. 2017 [14]	Poland	NR	2043	EURO, 2014	2431.8	$, 2017
Van Der Valk, 2016 [15]	The Netherlands	31%	1120	EURO, 2011	1459.3	$, 2017
Van Der Valk, 2014 [19]	The Netherlands	39%	1580^a^	EURO, 2011	2058.7^a^	$, 2017

^a^The study reports total productivity losses of 395.21€ per 3 months per patients

^b^Base year was not reported. It is assumed that base year correspond to publication year

## Discussion

The chronic nature of UC as well as the way the disease evolve over time makes it a costly condition to manage. Typically, costs of hospitalizations, surgery and the management of its complications are drivers of direct medical cost. Given the disease’s epidemiological characteristics and age distribution, the indirect costs due to productivity losses further contribute to high overall total disease costs. The objective of this review was to understand the impact of UC on indirect costs.

Understanding and quantifying indirect costs associated to biologics versus surgery was possible through one study by Van Der Valk published in 2015 [29], that compared indirect costs in patients under anti-TNF (*n* = 34) therapies with patients who underwent surgery, either J-pouch (*n* = 81) or Ileostomy (*n* = 48). Results indicated that indirect costs were highest in patients taking anti-TNF therapies (4340€ per patient year and 28 days of sick leave per year) compared to surgical patients (with 1508 $ per patient year and 20 days of sick leave per year for ileostomy and 1932 $ per patient year and 8 days of sick leave per year for pouch patients). However, little is known on the long-term cost trends of these interventions. In fact, surgery has typically been considered as a significant cost driver in UC patients, mainly due to the need for hospitalization as well as the risk for complications after the procedure. A recent review by Lindsay et al. [8] showed that, 5 years post-operation, the mean cost per patient with surgical complications was significantly greater compared to those without complications, representing 34, 714 $ additional costs per patient. As surgical complications represent a substantial burden in terms of cost of reoperation, physician fees, additional in-patient hospital stays and infertility treatment, further studies are needed to understand the direct and indirect cost of biologics versus surgery in UC.

As healthcare systems vary greatly between countries, it was expected to identify variations in indirect costs between Europe and the USA. Giving that the majorities of identified publications reported indirect costs in Europe, it was difficult to quantify this difference. However, we noted that costs related to absenteeism were greater in the USA compared to Europe. Productivity losses were only reported in three studies. Although limited information was found, it was possible to conclude that, on average, 35% of total UC costs were associated to indirect costs. This proportion remains uncertain, as indirect costs are very difficult to assess, mainly because of difficulties in measurement, especially when talking about presenteeism (i.e. the reduction of workers’ effectiveness due to illness).

This review allowed us to conclude that costs in UC tend to be highly variable based on the subpopulation to which they refer. In fact, Cohen et al. [18] reported that in the US, patients with UC have higher direct and indirect costs compared with matched controls [18]. In fact, patients with moderate to severe UC (*n* = 1728) had significantly (*p* < 0.0001) higher hospitalization rates (26.5% vs 6.2%) and adjusted total direct (23,085 $ vs 4932$) and indirect costs (5666 $ vs 1960 $) [18]. Similar conclusions were reached by Bodger et al. [30] and Hilson et al. [31] who reported significantly higher costs in severe patients compared to milder UC patients. Unfortunately, in the literature, indirect costs are poorly evaluated by UC disease state. Therefore, it remains also unclear whether these indirect costs are representative to the general UC population regardless of their disease severity/state.

This review has several limitations. No population-based studies with control patients were included in our analysis and our findings may not be applicable to all patients with UC. After completion of our analysis, results from a prospective study was published describing data for patients with UC and CD from the Danish national registry [32]. This population-based study reported no significant difference in indirect costs between IBD (UC and CD) and a control population. The authors attributed these results to a nationalized healthcare system, a high standard of treatment and a relatively young patient cohort. It was suggested that the indirect costs may increase in an older population, however this analysis was not conducted in the study [32].

Within the small number of studies that reported indirect costs in UC, heterogeneity of reporting data was a key limitation in analyzing and quantifying the impact of indirect costs in UC. Analysis was confounded by differences in costing components within the same category and across different cost categories, due to the lack of definition of these components. There remains uncertainty in the cost associated with absenteeism based on the limited information provided in the primary publications. A recent review by Kawalec et al. [14] concluded that IBD imposes a substantial personal burden and affects the ability to work, supporting our findings.

Costs were adjusted to the 2017 USD values to allow comparison between countries; however, these may not be comparable across different societies. Other limitations include: heterogeneity in the inclusion criteria (disease severity, disease history, patients’ characteristics).

## Conclusion

In conclusion, the findings of this review showed that indirect costs in UC are not well documented in the literature. Therefore, additional studies are needed in UC to quantify these costs per disease state and to evaluate the impact of the disease on patients’ functional abilities.

## Data Availability

All data generated or analyzed during this study are included in this published article.

## Abbreviations

DDW: Digestive Disease Week
ECCO: European Crohn’s and Colitis Organization
EMA: European Medicines Agency
FDA: Food and Drug Administration
PICOS: Population, Intervention, Comparator, Outcome, Study type
PRISMA: Preferred Reporting Items for Systematic Reviews and Meta-Analyses
RWE: Real World Evidence
UC: Ulcerative colitis
UEGW: United European Gastroenterology Week
USD: United States Dollar

## Footnotes

1 PICOS: Population, Intervention, Comparison, Outcome, Study type

2 The authors decided to focus on active UC as its when patients experience UC like symptoms.

3 Short/long-term disability is defined as work cessation through job loss, early retirement, or long-term absence due to being partially or fully disabled. According to Malinowski et al. [16] short-term disability entitles a person to claim rehabilitation benefits and long-term disability entitles a person to claim disability pension.

4 Total UC costs were defined as the sum of healthcare costs, productivity costs and out-of-pocket costs.
